# There is always glucose in normal urine: unspecific excretion associated with serum glucose and glomerular filtration rate

**DOI:** 10.1093/ije/dyac060

**Published:** 2022-04-04

**Authors:** Tianqi Li, Andrei Ihanus, Pauli Ohukainen, Marjo-Riitta Järvelin, Johannes Kettunen, Ville-Petteri Mäkinen, Tuulia Tynkkynen, Mika Ala-Korpela

**Affiliations:** Computational Medicine, Faculty of Medicine, University of Oulu, Oulu, Finland; Center for Life Course Health Research, Faculty of Medicine, University of Oulu, Oulu, Finland; Biocenter Oulu, University of Oulu, Oulu, Finland; Computational Medicine, Faculty of Medicine, University of Oulu, Oulu, Finland; Center for Life Course Health Research, Faculty of Medicine, University of Oulu, Oulu, Finland; Biocenter Oulu, University of Oulu, Oulu, Finland; NMR Metabolomics Laboratory, School of Pharmacy, University of Eastern Finland, Kuopio, Finland; Computational Medicine, Faculty of Medicine, University of Oulu, Oulu, Finland; Center for Life Course Health Research, Faculty of Medicine, University of Oulu, Oulu, Finland; Biocenter Oulu, University of Oulu, Oulu, Finland; Center for Life Course Health Research, Faculty of Medicine, University of Oulu, Oulu, Finland; Unit of Primary Health Care, Oulu University Hospital, OYS, Oulu, Finland; Department of Epidemiology and Biostatistics, MRC-PHE Centre for Environment and Health, School of Public Health, Imperial College London, London, UK; Department of Life Sciences, College of Health and Life Sciences, Brunel University London, UK; Computational Medicine, Faculty of Medicine, University of Oulu, Oulu, Finland; Center for Life Course Health Research, Faculty of Medicine, University of Oulu, Oulu, Finland; Biocenter Oulu, University of Oulu, Oulu, Finland; Department of Public Health and Welfare, Finnish Institute for Health and Welfare, Helsinki, Finland; Computational Medicine, Faculty of Medicine, University of Oulu, Oulu, Finland; Center for Life Course Health Research, Faculty of Medicine, University of Oulu, Oulu, Finland; Computational and Systems Biology Program, Precision Medicine Theme, South Australian Health and Medical Research Institute, Adelaide, Australia; Australian Centre for Precision Health, Unit of Clinical and Health Sciences, University of South Australia, Adelaide, Australia; Computational Medicine, Faculty of Medicine, University of Oulu, Oulu, Finland; Center for Life Course Health Research, Faculty of Medicine, University of Oulu, Oulu, Finland; Biocenter Oulu, University of Oulu, Oulu, Finland; NMR Metabolomics Laboratory, School of Pharmacy, University of Eastern Finland, Kuopio, Finland; Computational Medicine, Faculty of Medicine, University of Oulu, Oulu, Finland; Center for Life Course Health Research, Faculty of Medicine, University of Oulu, Oulu, Finland; Biocenter Oulu, University of Oulu, Oulu, Finland; NMR Metabolomics Laboratory, School of Pharmacy, University of Eastern Finland, Kuopio, Finland

Urinary glucose analytics has been compromised by methodological issues for a long time. Rudimentary problems in non-specific methods^1^^,^^2^ and the wide usage of glucose test strips (with detection limits as high as 5.6 mmol/L) have led to a situation in which it is commonly believed, and also taught in contemporary renal physiology textbooks,^3^ that normal human urine does not contain glucose. We encapsulate here—via modern nuclear magnetic resonance (NMR) spectroscopy^4^ experiments in two independent population cohorts—that this is an apparent misconception. These novel data are also put in an epidemiological context with a wide selection of customary clinical and biochemical measures.

Utilizing quantitative NMR spectroscopy^4^ we analysed overnight spot urine samples from two independent population cohorts: the Northern Finland Birth Cohort 1966 and 1986 with 4482 (age 46 years, 43% men) and 1010 (age 33 years, 42% men) participants, respectively. We were able to detect and quantify glucose in 99.1% of these 5492 urine samples and the concentration distributions were almost identical, slightly positively skewed with a median relative concentration of 24.2 and 22.5 µM per 1 mM creatinine in NFBC1966 and NFBC1986, respectively (Figure 1A). The concentrations appeared slightly higher for women, likely due to their lower muscle mass and thus lower concentrations of circulating creatinine, leading to lower amounts of excreted creatinine into the urine. Typical absolute glucose concentrations in urine were between 0.1 and 0.5 mmol/L.

**Figure 1:**
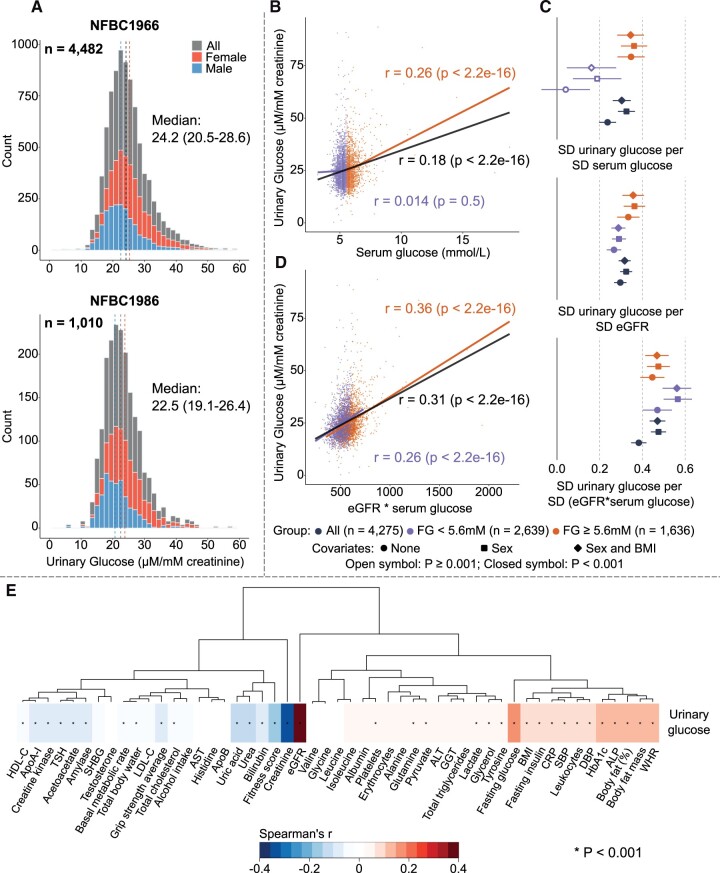
(A) Distribution of urinary glucose concentration in NFBC1966 (*n* = 4482) and NFBC1986 (*n* = 1010). Values of >60 μM/mM creatinine are not drawn for clarity (*n* = 106 in NFBC1966 and *n* = 7 in NFBC1986). (B)–(E) Correlation and regression analyses of urinary glucose in NFBC1966. In these analyses, *n* = 4275 due to the exclusion of values higher than eight times the interquartile range (*n* = 75), participants whose urinary glucose was below the detection limit (*n* = 36) and those for whom the serum fasting glucose was not available (*n* = 96). The correlation analyses of urinary glucose with (B) serum glucose and with (D) the multiplication of serum glucose and eGFR are depicted for all participants, for normoglycaemic participants (serum fasting glucose <5.6 mmol/L; *n* = 2639) and for those whose serum fasting glucose concentrations were ≥5.6 mmol/L (*n* = 1636). (C) Results from linear regression models for urinary glucose with serum glucose, eGFR or the multiplication of serum glucose and eGFR as the explanatory variable. The results for all participants, those who were normoglycaemic and those whose serum fasting glucose concentrations were ≥5.6 mmol/L are colour coded in (B)–(D) (black, blue and orange, respectively). (E) Partial rank correlations adjusted for sex to illustrate the associations between urinary glucose and 51 customary clinical and biochemical measures. The heat map is organized via column dimensional hierarchical clustering. In the analyses for both (C) and (E), the multiple comparison corrected *P*-value threshold of 0.001 was applied to denote evidence in favour of an association. ALP, alkaline phosphatase; ALT, alanine aminotransferase; ApoA-I, apolipoprotein A-I; ApoB, apolipoprotein B; AST, aspartate aminotransferase; BMI, body mass index; C, cholesterol; CRP, C-reactive protein; DBP, diastolic blood pressure; eGFR, estimated glomerular filtration rate (using the CKD-EPI formula); GGT, gamma−glutamyl transferase; HbA1c, glycated haemoglobin; HDL, high-density lipoprotein; LDL, low-density lipoprotein; SHBG, sex hormone binding globulin; SBP, systolic blood pressure; TSH, thyroid-stimulating hormone; WHR, waist-to-hip ratio.

Glucose is indispensable in energy metabolism and it would be comprehensible that all filtered glucose would be reabsorbed by the kidneys. The main processes of glucose reabsorption are rather well understood. Under normoglycaemia, sodium-glucose cotransporter SGLT2 in the early proximal tubule reabsorbs ∼97% of filtered glucose. The remaining 3% is reabsorbed by SGLT1 in the late proximal tubule.^5^ At high plasma glucose concentrations (>10 mmol/L) the tubular glucose reabsorption is known to saturate, triggering a pronounced part of filtered glucose to be excreted into the urine.

Most of the participants studied here were normoglycaemic (serum fasting glucose <5.6 mmol/L) and their kidneys were thus far from saturated with respect to glucose reabsorption. Accordingly, no association was detected between serum and urinary glucose. However, when serum fasting glucose values were ≥5.6 mmol/L, an association emerged (Figure 1B). The latter can perhaps be linked to early phases of saturation in glucose reabsorption, but the coherent small concentrations of urinary glucose in normoglycaemia call for another explanation.

Figure 1C depicts three regression analyses with urinary glucose: serum glucose, estimated glomerular filtration rate (eGFR using the CKD-EPI formula) and the multiplication of serum glucose and eGFR. All associations are positive, suggesting that urinary glucose concentrations are affected by circulating glucose as well as eGFR. Consistently with Figure 1B, the association between urinary and serum glucose is very weak for normoglycaemic individuals. However, the association of eGFR with urinary glucose is rather coherent for all serum glucose concentrations. This results in the strongest association between urinary glucose and the multiplication of serum glucose and eGFR, with a unanimous trend for all serum glucose concentrations as demonstrated also by the correlation plot in Figure 1D.

The physiological basis for the molecular content in the morning spot urine samples is filtration and accumulation in mostly fasting conditions overnight. Thus, the analyses were done with categories based on only fasting serum glucose concentrations. Despite the anticipated reality that the metabolic data for spot urine samples are biologically scattered, accounting for the eGFR clearly elicits the connection between circulating glucose and urinary glucose. This finding intrinsically suggests that quantitative urinary concentrations of glucose may contain advantageous information on kidney function and disease risk at the population level.

We would interpret these findings as small deficiencies in the combined function of SGLT2 and SGLT1. As the kidneys filter the entire plasma volume some 60 times daily, it is not unexpected that some glucose molecules could escape the reabsorption by SGLT2 and SGLT1, though the process is known to be highly effective.^5^ This would lead to unspecific excretion of glucose into the urine also under normoglycaemia.

The epidemiological data do not allow gauging how gluconeogenesis or other molecular mechanisms in the kidneys potentially affect the urinary concentrations of glucose. In addition, neither potential change in kidney function related to long-term conditions (e.g. diabetes) nor instant systemic conditions (e.g. meal induced hyperglycaemia) can be presumed based on the current data. Nonetheless, these direct quantitative molecular data via NMR spectroscopy unequivocally show that glucose is always present also in the urine of normoglycaemic individuals. This is a fundamental issue that should be acknowledged and corrected in elementary teaching materials.

Furthermore, Figure 1E shows novel associations of urinary glucose with a wide selection of customary clinical and biochemical measures at the population level. For example, the urinary concentration of glucose was positively associated with body mass and composition (e.g. waist-to-hip ratio, body mass index, body fat mass and body fat percentage), diabetes risk factors (fasting insulin and glycated haemoglobin) and blood pressure. Negative associations with the urinary concentration of glucose were seen with fitness indicators (grip strength, fitness score and body water content), with some metabolic enzymes (amylase and creatine kinase) and with thyroid-stimulating hormone. High-density lipoprotein cholesterol and apolipoprotein A-I also associated negatively with urinary glucose, but no associations were seen with low-density lipoprotein cholesterol, triglycerides or apolipoprotein B. Alkaline phosphatase was positively associated with urinary glucose, but the other commonly measured liver enzymes (gamma−glutamyl transferase, alanine and aspartate aminotransferase) did not show any association. These population level associations with urinary glucose concentrations with various clinical and biochemical indicators, revealed here for the first time, appear logical and suggest that the unspecific excretion of glucose into the urine is likely to be of epidemiological, maybe even translational, interest.

The present newly introduced methodology—feasible in large-scale epidemiology and clinical studies^4^—thus opens a novel possibility to study completely unexplored urinary glucose concentrations at the population level and whether they would provide any new insight on kidney function and/or disease risk.

## Ethics approval

The Ethics Committee of the Faculty of Medicine, University of Oulu has approved NFBC1986 (17.6.1999) and NFBC1966 studies (17.6.1996). In addition, the Ethics Committee of the Northern Ostrobothnia Hospital District has approved NFBC1966 (94/2011) and NFBC1986 (108/2017). All clinical investigations were conducted according to the principles expressed in the Declaration of Helsinki. All participants gave written informed consent.

## Data Availability

The NFBC data used in the current study are available through an application process for researchers who meet the criteria to access confidential data: https://www.oulu.fi/nfbc/.
